# Validation of a commercially available anti-REDD1 antibody using RNA interference and REDD1-/- mouse embryonic fibroblasts

**DOI:** 10.12688/f1000research.7691.1

**Published:** 2016-03-01

**Authors:** Deborah L. Grainger, Lydia Kutzler, Sharon L. Rannels, Scot R. Kimball

**Affiliations:** 1 Proteintech Group, Manchester, UK; 2 Department of Cellular and Molecular Physiology, Penn State University College of Medicine, Hershey, PA, USA

**Keywords:** DDIT4, mTOR, REDD1, thapsigargin

## Abstract

REDD1 is a transcriptional target gene of p53 and HIF-1, and an inhibitor of mTOR (mechanistic target of rapamycin) complex 1 (mTORC1)-signaling through PP2A-dependent interaction, making it an important convergence point of both tumor suppression and cell growth pathways. In accordance with this positioning, REDD1 levels are transcriptionally upregulated in response to a variety of cellular stress factors such as nutrient deprivation, hypoxia and DNA damage. In the absence of such conditions, and in particular where growth factor signaling is activated, REDD1 expression is typically negligible; therefore, it is necessary to induce REDD1 prior to experimentation or detection in model systems. Here, we evaluated the performance of a commercially available polyclonal antibody recognizing REDD1 by Western blotting in the presence of thapsigargin, a pharmacological inducer of ER stress well known to upregulate REDD1 protein expression. Further, REDD1 antibody specificity was challenged in HEK-293 cells in the presence of RNA interference and with a REDD1 ^-/-^ mouse embryonic fibroblast knockout cell line. Results showed reproducibility and specificity of the antibody, which was upheld in the presence of thapsigargin treatment. We conclude that this antibody can be used to reliably detect REDD1 endogenous expression in samples of both human and mouse origin.

## Introduction

Mechanistic target of rapamycin complex 1 (mTORC1) is a central signaling node in the cellular response to nutrient availability and growth factor signaling, initiating and regulating processes such as protein synthesis, ribosome biogenesis and *de novo* lipogenesis when active (reviewed by Laplante and Sabatini ^1^). However, in times of amino acid deprivation or energy deficit, the cell must switch from these anabolic processes to maintain energy homeostasis. The switch is controlled by changes in mTORC1 activity that occur in response to variations in the availability of nutrients and energy requirements of the cell (see recent review by Albert and Hall ^2^).

REDD1 (protein regulated in development and DNA damage response 1) also known as DDIT4 (DNA damage-inducible transcript 4 protein) or RTP801, is a 232 amino-acid upstream repressor of mTORC1 activity ^3– 5^ that is transcriptionally upregulated by growth factor signaling and in response to amino acid deprivation, among other stimuli. The mechanism by which REDD1 acts to repress mTORC1 signaling has been under investigation for almost a decade ^6^. These studies are focused on REDD1’s suppression of mTORC1 via its stimulation of the tuberous sclerosis complex 2 (TSC2); however, the method by which REDD1 activates TSC2 remained elusive ^6^.

Recent data have revealed a model where REDD1 promotes the association of protein phosphatase 2A (PP2A) with serine/threonine-protein kinase Akt, leading to the dephosphorylation of the kinase on Thr ^308^ ^6^. This dephosphorylation of Thr ^308^ (but not the Ser ^473^ residue) subsequently leads to a reduction in the Akt-mediated phosphorylation of TSC2, followed by TSC2-mediated stimulation of Rheb GTPase activity. This results in accumulation of Rheb in the GDP bound form, and thus the inhibition of mTORC1 activity.

Despite its ubiquitous distribution, expression of REDD1 is typically negligible in developed tissues until cells encounter conditions of nutrient and energy deprivation ^7, 8^ or – particularly in the case of skeletal muscle tissue – during endurance exercise ^9^ when energy is prioritized for movement. It is also transcriptionally activated by p53 ^10^ hypoxia inducible factor-1 (HIF-1) ^11^ and ATF4 ^12^ transcription factors, in accordance with initial findings that REDD1 levels increase in response to a variety of cellular stresses, including hypoxia ^3^, ER stress ^12^ and by agents that cause DNA damage such as UV radiation ^13^. Several pharmacological agents can also be used to induce REDD1 expression in experimental models where REDD1 follows endogenous patterns of expression. Agents such as the glucocorticoid dexamethasone ^14^ and the non-competitive inhibitor of the sarco/endoplasmic reticulum Ca2+ ATPase (SERCA) pump thapsigargin ^12^ have been shown to upregulate expression of REDD1.

We examined Proteintech’s anti-REDD1 antibody (catalog number 10638-1-AP) in scenarios where REDD1 levels were either reduced or absent. The antibody, which is raised against a whole-fusion protein immunogen consisting of amino acid residues 1-232, is cited in over 90% of publications utilizing REDD1 antibodies (CiteAb: http://www.citeab.com/search?q=REDD1), and a total of 110 publications (at the time of writing, http://www.ptglab.com/Products/REDD1-Antibody-10638-1-AP.htm?publication=1). REDD1 reduction was achieved using RNA interference (RNAi). Antibody specificity was further supported by Western blotting experiments performed using samples from a REDD1 ^-/-^ knockout mouse embryonic fibroblast (MEF) cell line, and controlled in the presence of thapsigargin.

## Materials and methods

### Antibody details

The anti-REDD1 antibody used in this study (Cat#: 10638-1-AP, RRID: AB_2245711) is a rabbit polyclonal antibody made exclusively by Proteintech Group. It was raised against a fusion protein corresponding to the full-length human REDD1-protein sequence (amino acids 1-232). Full sequence: MPSLWDRFSSSSTSSSPSSLPRTPTPDRPPRSAWGSATREEGFDRSTSLESSDCESLDSSNSGFGPEEDTAYLDGVSLPDFELLSDPEDEHLCANLMQLLQESLAQARLGSRRPARLLMPSQLVSQVGKELLRLAYSEPCGLRGALLDVCVEQGKSCHSVGQLALDPSLVPTFQLTLVLRLDSRLWPKIQGLFSSANSPFLPGFSQSLTLSTGFRVIKKKLYSSEQLLIEEC.

All validations in this study were undertaken using lot# 00019207.

A protein BLAST search demonstrates that the full-length sequence of human REDD1 shares over 90% homology with mouse Redd1.

Goat anti-Rabbit polyclonal secondary antibody, conjugated with horseradish peroxidase (HRP) was obtained from Jackson ImmunoResearch (Cat# 111-035-003, RRID: AB_2313567). This antibody is specific for both the heavy and light chains of Rabbit IgG, and was affinity purified to reduce cross-reactivity to other species.

The anti-α-tubulin antibody used in this study (sc-32293, RRID: AB_628412) is a mouse monoclonal antibody purchased from Santa Cruz Biotechnology, Inc. The antibodies used in this study are summarised in Table 1.

**Table 1.** Antibodies used and their manufacturer details.

Antibody	Manufacturer	Cat. No.	RRID
Anti-REDD1 Rabbit Polyclonal	Proteintech	10638-1-AP	AB_2245711
Anti-α-tubulin Mouse Monoclonal (Clone:DM1A)	Santa Cruz Biotechnology	Sc-32293	AB_628412
Peroxidase AffiniPure Goat Anti-Rabbit IgG (H+L)	Jackson ImmunoResearch	111-035-003	AB_2313567

### Cell lines

HEK-293 cells were purchased from the American Type Culture Collection (ATCC CRL-1573) and used in shRNA knock down validation studies of the 10638-1-AP anti-REDD1 antibody.

The REDD1 ^-/-^ knockout MEF cell line and permission for its use was obtained from Dr. Leif Ellisen. Construction of the REDD1 ^-/-^ allele and generation of this cell line was previously described by Sofer *et al.* ^1^.

DMEM high-glucose medium was purchased from Gibco/Life Technologies (11965-092) and Fetal Bovine Serum Premium Select was purchased from Atlanta Biologicals (51150).

### shRNA plasmid design

The target site for REDD1 knock down was determined by available literature and online shRNA design tools (Broad Institute: http://www.broadinstitute.org/rnai/public/seq/search, siDirect version 2.0: http://sidirect2.rnai.jp/). We designed two single-stranded 21mer stem DNA oligonucleotides, encoding the target siRNA (sense strand), in addition to their single-stranded complementary strand counterparts (antisense strand). The two sequences (sense and antisense) were linked together by a short linker sequence TTCAAGACG that forms a hairpin loop structure upon sequence expression. At the end of the shRNA template we added a 6 nucleotide poly (T) tail, recognized as a termination signal. The 5’ ends of the two oligonucleotides are non-complementary and form the BamHI and HindIII restriction site overhangs which facilitated directional cloning into the pGenesil-1 vector. pGenesil-1 is a plasmid vector modified by addition of the hU6 promoter to the pEGFP-C1 plasmid. The sense strand sequences of both shRNA constructs designed for this study are shown in Table 2.

**Table 2.** Sense strand sequences of the shRNA constructs.

shRNA sequence identifier	Sequence
REDD1-1	GTGTAGCATGTACCTTATTAT
REDD1-2	ACACCTGGCAGCTGCGTTTAA
Control	ACTACCGTTGTTATAGGTGT

### RNAi knock down of REDD1

HEK-293 cells (2×10 ^5^) were seeded in fresh, full media in duplicate per experimental condition in 12-well CellBind plates (Corning). Cells were incubated for 24 hours at 37°C in a humidified incubator (5% CO _2_) prior to REDD1 knock down. Lipofectamine 2000 (Life Sciences, #11668027), a cationic lipid based transfection reagent, was mixed with pGenesil-1 vector containing REDD1 shRNA or control shRNA construct at a ratio of 1:1 in reduced serum medium and incubated for 15 minutes at 37°C to allow formation of lipid-DNA complexes. Cell culture media in 12-well plates were replaced with fresh pre-warmed media lacking serum before addition of lipid-DNA complex to HEK-293 cells. Cells were incubated with lipid-DNA complex for 48 hours at 37°C (5% CO _2_), before removal of media and washing in PBS. Cells were then harvested and lysed by application of 1X Laemmli sample buffer and scraping. REDD1 levels were detected by Western blot experiment.

### Thapsigargin treatment

REDD1 ^-/-^ and wild type cells were seeded at 2.5×10 ^5^ in 12 well dishes in DMEM high glucose media containing 10% fetal bovine serum and 1% penicillin/streptomycin (Life Technologies #15070-063). Cells were incubated for at least 24 hours at 37°C in a humidified incubator with 5% CO _2_ prior to treatment. The medium was removed and replaced with medium containing either 100 nM thapsigargin (Sigma #T9033) or vehicle (ethanol), and the cells were returned to the incubator for 4 hours. The medium was then removed, cells were washed once with cold PBS, and harvesting and lysis by application of 1X Laemmli sample buffer and scraping.

### Western blotting

Cell lysates were heated at 100°C for 5 minutes, before 40 µl lysate (per sample) was loaded onto a 4–20% Criterion SDS-polyacrylamide gel (BioRad). Proteins were separated by SDS-PAGE (200V for 1 hour). Separated proteins were transferred to PVDF membrane in a Criterion Blotter (BioRad) at 50V for 90 min. Membrane was blocked in 5% milk in TBS-Tween (TBS-T) for one hour before addition of primary antibody (1:800 dilution) in 1% milk TBS-T. Primary antibody incubation was carried out overnight before membrane washing in TBS-T and addition of secondary antibody (1:7,500 dilution) in 5% milk TBS-T for a further hour. Excess secondary antibody was removed by washing in TBS-T, before Protein bands were visualised by incubation with Clarity Western ECL Blotting Substrate (BioRad) and imaging on a FluorChem M imaging system (ProteinSimple). All steps were carried out at room temperature and are listed Table 3. Western blot signals were also further analysed by densitometry analysis using Alphaview (ProteinSimple).

**Table 3.** Experimental protocol and reagents used.

Protocol steps	Reagent(s)	Time (mins/)
Transfer	Towbin buffer with 20% methanol (25 mM Tris-base, 192 mM Glycine, 20% methanol)	90
Blocking	5% milk TBS-Tween (TBS-Tween: 20 mM Tris-base, 150 mM NaCl, 50 mM KCl, 0.2% Tween-20)	60
Primary antibody incubation	Primary antibody - 10638-1-AP (1:800) 1% milk TBS-Tween	Overnight
Washing	TBS-Tween: 20 mM Tris-base, 150 mM NaCl, 50 mM KCl, 0.2% Tween-20	(3 x) 5
Secondary antibody incubation	Peroxidase AffiniPure Goat Anti-Rabbit IgG (1:7,500) 5% milk TBS-Tween	60

## Results

**Figure 1.**
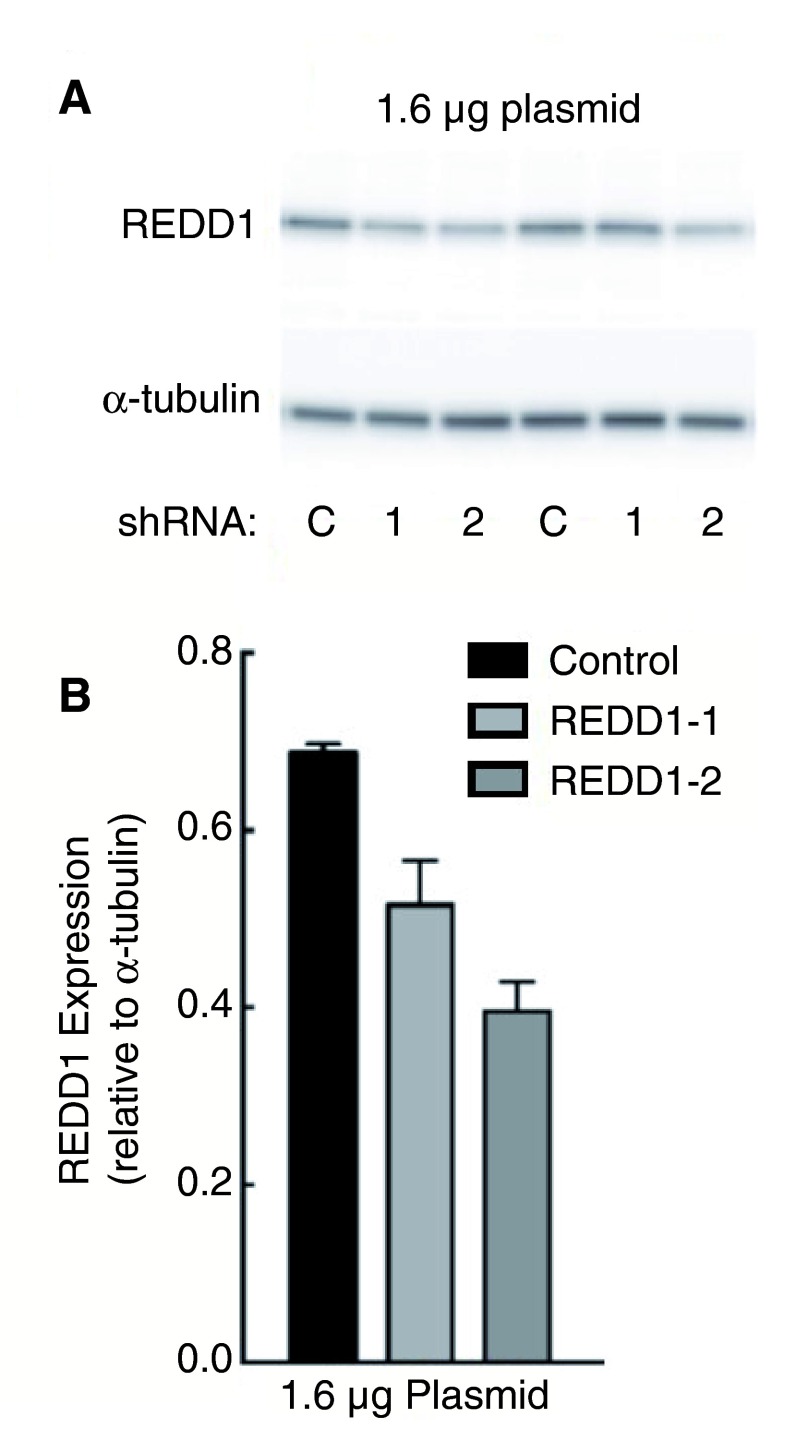
HEK-293 cells were transfected with either 1.6 µg REDD1-1 (1), REDD1-2 (2) or control (C) shRNA plasmid and incubated for 48 hours before subsequent cell lysis and Western blotting. REDD1 was detected using Proteintech’s 10638-1-AP anti-REDD1 antibody ( **A**). Signal intensity was measured by densitometry analysis and blotted relative to α-tubulin control signal, n=2 ( **B**).

**Figure 2.**
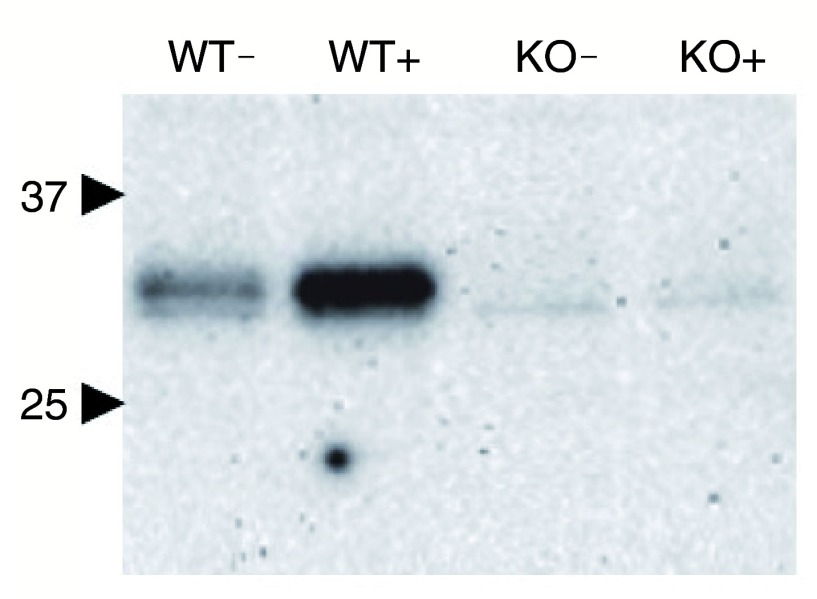
REDD1 ^+/+^ (WT) and REDD1 ^-/-^ knockout (KO) MEF cells were incubated with thapsigargin (+) or carrier control (-) before lysate preparation and subsequent Western blotting with the anti-REDD1 antibody from Proteintech. REDD1 bands were indirectly detected using Proteintech’s 10638-1-AP anti-REDD1 antibody (n=3). Membrane exposure = 20 minutes. Numbers and block arrows indicate positions of 37 kDa and 25 kDa bands of the MW marker.

Raw data for ‘Validation of a commercially available anti-REDD1 antibody using RNA interference and REDD1 ^-/-^ mouse embryonic fibroblasts’ This dataset includes uncropped blots ( Figure 1a, Figure 2) and signal intensities ( Figure 1b). Click here for additional data file. Copyright: © 2016 Grainger DL et al. 2016 Data associated with the article are available under the terms of the Creative Commons Zero "No rights reserved" data waiver (CC0 1.0 Public domain dedication).

### REDD1 specific RNAi treated cells show a reduction of protein level on Western Blotting

We reduced endogenous levels of REDD1 using a homemade shRNA construct designed to target REDD1 mRNA (as detailed in the methods section). An initial RNAi experiment was carried out using 1.6 µg REDD1 shRNA plasmid or control shRNA plasmid to transfect HEK-293 cells. Cells were incubated with shRNA plasmid for 48 hours before cell lysis. REDD1 levels were then immediately visualized by Western blot experiment using the Proteintech anti-REDD1 antibody (10638-1-AP).

We found that REDD1 expression was reduced by up to 58% in cells incubated with REDD1-targeting shRNA (this percentage was obtained using data from REDD1-2 shRNA samples, n=2) compared to cells transfected with the control plasmid as shown in Figure 1a (assessed by densitometry measurements normalised to alpha-tubulin levels, as shown in Figure 1b). To further observe whether a higher percentage of reduction could be achieved by increasing the amount of shRNA, a second experiment using 3.2 µg REDD1 shRNA plasmid was performed. However, a further reduction of REDD1 levels was not observed (results not shown).

### REDD1 ^-/-^ knockout MEF cell line further demonstrates antibody specificity

A second experiment was set up to further probe the specificity of Proteintech’s REDD1 antibody. REDD1 ^-/-^ knockout and REDD1 ^+/+^ MEF cells were incubated with thapsigargin or carrier control before protein lysate preparation and subsequent Western blotting.

In accordance with Proteintech’s REDD1 antibody being highly specific for REDD1, the Western blot results show that REDD1 signal is present only in REDD1 ^+/+^ MEF cells and increase in response to thapsigargin. No such response was seen in the REDD1 ^-/-^ knockout MEFs regardless of thapsigargin treatment. This observation was seen in three independent experiments (n=3); the data shown in Figure 2 are representative and were obtained following a 20 minute exposure of the Western blot membrane.

## Conclusion

Through testing Proteintech’s anti-REDD1 antibody (10638-1-AP) in a REDD1 ^-/-^ knockout (KO) MEF cell line, and to a certain extent, by using it in combination with REDD1 shRNA plasmids in HEK-293 cells, we have demonstrated a loss or reduction of REDD1 protein signal on Western blot membranes, respectively – indicative of antibody target specificity. Antibody signal also responds accordingly to the presence of thapsigargin-treated MEF cell lines. This paper validates the specificity of this commercially available antibody for the REDD1 protein in Western blots, supporting a significant prior body of work that has utilised this immunological reagent.

There are several observations we must address in this conclusion; the first being REDD1’s observed molecular weight (MW) on Western blot membranes. Despite having a predicted MW of 25 kDa, REDD1 often migrates around 35 kDa during SDS-PAGE due to the presence of multiple lysines in its amino acid sequence, as documented by other sources ^10, 16, 17^. The MW seen for REDD1 in our investigations are in concordance with the reported MW shift. Second, the faint bands present in Figure 2 could perhaps warrant further investigation, but as the blot shown represents a prolonged membrane exposure (20 minutes), these artefacts are unlikely to cause misinterpretation of results in experiments, given the signal intensity in the stimulated wild type control (WT+).

The level of REDD1 knock down in the RNAi experiment was lower than desired (calculated to be around a 58% reduction of REDD1 levels). However, given the loss of REDD1 signal in the Western blots featuring REDD1 ^-/-^ knockout (KO) MEF cells – a cell line well-characterised by previous studies ^6, 15, 18– 20^ – using the same antibody lot as in the knock down experiment, we feel there is enough evidence to confirm REDD1 specificity.

At the time of paper submission, the #00019207 lot was the only lot available from the vendor. Lot-to-lot testing is carried out by the vendor, but not yet in the context of REDD1 absence or knock down (though data of an initial RNAi experiment appear on the online datasheet, they represent the same lot #00019207); therefore it would be interesting to follow up this study in future by testing further lots of the 10638-1-AP REDD1 antibody in such settings where REDD1 levels are absent or compromised.

## Data availability

The data referenced by this article are under copyright with the following copyright statement: Copyright: © 2016 Grainger DL et al.

Data associated with the article are available under the terms of the Creative Commons Zero "No rights reserved" data waiver (CC0 1.0 Public domain dedication). 

*F1000Research*: Dataset 1. Raw data for ‘Validation of a commercially available anti-REDD1 antibody using RNA interference and REDD1 ^-/-^ mouse embryonic fibroblasts’, 10.5256/f1000research.7691.d114991 ^21^
