# Supplementary material for: Validation of a commercially available anti-REDD1 antibody using RNA interference and REDD1-/- mouse embryonic fibroblasts
Source: F1000Res. 2016 Mar 1;5:250. [Version 1] doi: 10.12688/f1000research.7691.1 (PMC4893971; doi:10.12688/f1000research.7691.1)
Supplement: Peer review history

# Peer review: Referee response for version 1

**DOI:** 10.5256/f1000research.8281.r12892

**Published:** 2016-03-15

**Authors:** Mei Yee Leung

**Affiliations:** 1 PeproTech EC Ltd, London, UK

This article by Deborah L. Grainger *et al*, clearly documents the validation of Rabbit anti-Human REDD-1 in a Western blot application. This data has further reinforced the quality of this already well established and cited commercial antibody by demonstrating specificity using RNAi technology and knock out cell lines. The paper has quite conclusively shown that Proteintech Rabbit anti-Human REDD-1, catalogue number 10638-1-AP, Lot #00019207 is an antibody that binds to a specific region of the human REDD-1 full length protein.

However, I have the following comments regarding some potential questions this article may generate. Addressing these would mostly likely strengthen this antibody validation paper and allow the reader to better able to make an informed choice when selecting the right anti-REDD-1 antibody.

1. Since the paper addresses only application in Western blot, could this be included in the title?
2. Many tested applications are listed for this antibody, Catalogue No. 10638-1 in the datasheet including IHC, could the validation for this antibody be extended to include ICC? It would be interesting to see whether thapsigargin induced expression of REDD-1 in Western blot is corroborated by the same in ICC.
3. Figure 2. REDD-1 expression in WT- is quite substantial visually. Would you expect this level of expression under physiological 02?
4. Following on from point 3, REDD-1 is only expressed under certain environmental conditions, including hypoxia [reference 10, Ellisen LW et al, 2002], and the latter has been widely used to induce REDD-1 expression in other studies. Some clarification as to why HEK-293 cells cultured in 5%C02 is showing expression of REDD-1 is warranted and should be included in the discussion.
5. Can you explain the choice of using HEK 293 cell line? Also, was there any reason why the other cell lines listed as positive controls in the datasheet wasn’t included?
6. The probability (high or low) of this anti-REDD-1 (DDIT4 -DNA-Damage-Inducible Transcript 4) antibody cross reacting with REDD-2 protein (DDIT4L -DNA-Damage-Inducible Transcript 4-Like) should also discussed.
7. Will there be any consideration for epitope mapping to be implemented in future lots?

I have read this submission. I believe that I have an appropriate level of expertise to confirm that it is of an acceptable scientific standard.

# Peer review: Referee response for version 1

**DOI:** 10.5256/f1000research.8281.r12690

**Published:** 2016-03-08

**Authors:** Peter Cavnar

**Affiliations:** 1 Department of Biology, University of West Florida, Pensacola, FL, USA

The paper titled “Validation of a commercially available anti-REDD1 antibody using RNA interference and REDD1-/- mouse embryonic fibroblasts” shows convincing data that demonstrates the specificity of the commercially available anti-REDD1 rabbit polyclonal antibody from Proteintech, Inc. (Cat. No. 10638-1-AP, lot #00019207). I have some minor concerns that would help strengthen this report if addressed:

Concerns:

1. Figure 1 shows data from two replicates using two separate shRNAs targeted to REDD1. This experiment should be replicated at minimum three times. Furthermore, looking at the raw gels can the authors clarify that these two replicates are from two separates sets of HEK cultures and transfections although they were run on a single gel? What are the other lanes in the raw images?
2. Do the authors see any differences in specificity and signal intensities when blocking with BSA as compared to 5% milk?
3. Is the knockdown quantified in Figure 1B statistically significant?
4. Including a protein standard on the gel to emphasize the molecular weight disparity of REDD1 on SDS-PAGE would be helpful. Do the authors observe this migration of 35 kDa compared to the predicted 25 kDa in both the MEFs and HEKs cell lysates?
5. What protein standard (source) was used on these gels that can be observed in the raw data set?
6. Have the authors tried doing a secondary only control? This could help determine if the band they observe in Figure 2 KO cells is being caused by the secondary antibody.
7. Figure 2 says that n=3 however only two gels are shown in the raw image sets. Can the authors include the third gel?
8. Figure 2 raw data set shows two replicates that look like they are from the same blot. Are these from separate cell lysates and Western blots or was were these two replicates completed simultaneously?

I have read this submission. I believe that I have an appropriate level of expertise to confirm that it is of an acceptable scientific standard.
